# ATG4B and pS383/392-ATG4B serve as potential biomarkers and therapeutic targets of colorectal cancer

**DOI:** 10.1186/s12935-023-02909-7

**Published:** 2023-04-10

**Authors:** Wan-Hsiang Hu, Ting-Ting Liu, Pei-Feng Liu, Paul Morgan, I-Ling Lin, Wei-Lun Tsai, Yi-Yun Cheng, Ang-Tsen Hsieh, Tsung-Hui Hu, Chih-Wen Shu

**Affiliations:** 1 Department of Colorectal Surgery, Kaohsiung Chang Gung Memorial Hospital, Chang Gung University College of Medicine, Kaohsiung, 83341 Taiwan; 2 Graduate Institute of Clinical Medical Science, College of Medicine, Chang Gung University, Kaohsiung, 83341 Taiwan; 3 Department of Pathology, Kaohsiung Chang Gung Memorial Hospital, Chang Gung University College of Medicine, Kaohsiung, 83341 Taiwan; 4 Department of Biomedical Science and Environmental Biology, Kaohsiung Medical University, Kaohsiung, 80708 Taiwan; 5 Institute of Biomedical Sciences, National Sun Yat-sen University, Kaohsiung, 80424 Taiwan; 6 Icahn School of Medicine at Mount Sinai, New York, NY 10029 USA; 7 Department of Medical Laboratory Science and Biotechnology, Kaohsiung Medical University, Kaohsiung, 80708 Taiwan; 8 Department of Laboratory Medicine, Kaohsiung Medical University Hospital, Kaohsiung, 80708 Taiwan; 9 Department of Internal Medicine, Kaohsiung Veterans General Hospital, Kaohsiung, 81362 Taiwan; 10 Innovative Incubation Center, Praexisio Taiwain Inc, National Tsing Hua University, Hsinchu, 30013 Taiwan; 11 Institute of Biopharmaceutical Sciences, National Sun Yat-sen University, No. 70, Lianhai Rd., Gushan Dist, Kaohsiung, 80424 Taiwan; 12 Division of Hepato-Gastroenterology, Department of Internal Medicine, Kaohsiung Chang Gung Memorial Hospital, Chang Gung University College of Medicine, Kaohsiung, 83301 Taiwan; 13 Center of Excellence for Metabolic Associated Fatty Liver Disease, National Sun Yat-sen University, Kaohsiung, 80424 Taiwan

**Keywords:** ATG4B, Phosphorylation, Prognosis, cancer

## Abstract

**Background:**

Autophagy related protease 4B (ATG4B) is a protease required for autophagy processing, which is strongly implicated in cancer progression.  Phosphorylation of ATG4B is crucial for activation of its protease activity.  However, little is known about the relationship of ATG4B and its phosphorylated form at Ser 383 and 392 sites (pS383/392-ATG4B), with clinical outcomes, particularly in colorectal cancer (CRC).

**Methods:**

The *ATG4B* gene expression in CRC patients was obtained from The Cancer Genome Atlas (TCGA) database to analyze its clinical relevance. Tissue microarrays composed of 118 CRC patient specimens were used to determine the associations of ATG4B and pS383/392-ATG4B protein levels with prognosis. The biological functions of ATG4B in CRC cells were inspected with cell proliferation, mobility and spheroid culture assays.

**Results:**

*ATG4B* gene expression was elevated in tumor tissues of CRC patients compared to that in adjacent normal tissues and high level of *ATG4B* expression was associated with poor survival. Similarly, protein levels of ATG4B and pS383/392-ATG4B were highly correlated with worse overall survival and disease-free survival. Stratification analysis results showed that high level of ATG4B had significantly higher risk of mortality in males and elderly patients compared to those female patients and patients 60 years or younger. In contrast, multivariate Cox’s regression analysis indicated that high level of pS383/392-ATG4B was significantly linked to unfavorable overall survival and disease-free survival of males and elderly patients, whereas, it had no correlation with female patients and patients 60 years or younger. Moreover, high level of ATG4B was positively associated with increased mortality risk in patients with advanced AJCC stages (III and IV) and lymph node invasion (N1 and N2) for both overall survival and disease-free survival. Nevertheless, high level of pS383/392-ATG4B was positively correlated with increased mortality risk in patients with early AJCC stages (I and II) and without lymph node invasion (N0). In addition, silencing ATG4B attenuated migration, invasion, and further enhanced the cytotoxic effects of chemotherapeutic drugs in two and three-dimensional cultures of CRC cells.

**Conclusions:**

Our results suggest that ATG4B and pS383/392-ATG4B might be suitable biomarkers and therapeutic targets for CRC.

**Supplementary Information:**

The online version contains supplementary material available at 10.1186/s12935-023-02909-7.

## Introduction

Colorectal cancer (CRC) has grown to become one of the most prevailing malignancies worldwide. Current epidemiology projections estimate that the diagnosis rate of CRC will surge beyond 50% by 2030 [1]. Furthermore, the 5-year survival rate for CRC patients who are diagnosed as late stage is below 15%, highlighting an urgent need to identify theranostic markers for early detection and treatment [2]. Various genetic mutations have been implicated in the development and progression of CRC patients correlating with poor outcomes. The most frequent mutation genes in CRC are kirsten rat sarcoma viral oncogene (KRAS, ranging 40–52%), TP53 (around 40–50%) and adenomatous polyposis coli (APC, ranging 30–70%) [3, 4]. These genetic mutations have been linked to tumorigenesis and progression of CRC. A current hypothesis suggests that there is an initial mutation in the APC gene which triggers the activation of β-catenin and promotes cell proliferation [5]. TP53 and KRAS are subsequent mutations that further promote cancer development and malignancy [6]. However, therapeutic strategies that target these key mutations are limited.

Autophagy is a cellular pathway to degrade and recycle components for normal homeostasis, which is implicated in many diseases, particularly cancer [7–10]. Autophagy can play a tumor suppressor role to prevent cancer, but it can also act as tumor promoter by facilitating the survival of cancer cells under nutrient/oxygen deprived stress conditions [10–13]. Autophagy allows for the degradation of mutated TP53 to inhibit tumor formation, while genetic and pharmacological inhibition of autophagy reduce tumor growth and invasiveness in KRAS mutated cancer cells in vivo [14–16]. Hypoxia and nutrient induce autophagy facilitate cancer survival via regulation of HIF-1 transcription and AMPK post-translation modification during tumor progression [13]. To date, the role of autophagy in CRC is still largely controversial. Cho et al. demonstrated in a small cohort (N = 40) that ATG4B levels were significantly reduced in tumor tissues compared to corresponding normal tissue [17]. Conversely though, elevated levels of ATG5 was correlated with worse overall survival and disease-free survival (N = 118) [18]. Furthermore, Koustas et al. showed that higher Beclin-1 protein levels are correlated with worse overall survival in CRC patients who were administered chemotherapy [19].

LC3 dot-like immunostaining is correlated with shorter survival in KRAS mutated CRC, whereas high cytoplasmic p62 is negatively associated with poor survival in KRAS mutated CRC [20]. However, the clinical association of the other autophagy-related (ATG) proteins with CRC is still largely unknown. ATG4B is an essential protease that cleaves proLC3 or lipidated LC3-II for appropriate autophagosome formation [21]. Previous studies have demonstrated that ATG4B expression is increased and associated with poor prognosis in Chronic Myeloid Leukemia (CML), breast cancer, and oral cancer [22–24]. In addition, phosphorylation of ATG4B at Ser 383/392 increases its protease activity, which is involved in cancer cell growth [25]. Gene silencing of ATG4B, or, inhibiting ATG4B activity diminishes cancer cell viability and sensitizes cancer cell to chemotherapeutic drugs in vitro and in vivo suggesting ATG4B might play a role as tumor promoter [22, 26–29]. However, the relationship between ATG4B or phosphorylated ATG4B and clinical outcome in patients with CRC is not known.

To have a better understanding of the clinical correlation of ATG4B in CRC patients, we compared the protein levels of ATG4B and its phosphorylated form at Ser383/392 (pATG4B) with clinical outcomes in patients with CRC. We found that both ATG4B and pS383/392-ATG4B were connected to increasing severity of CRC. Furthermore, different demographic and clinicopathologic factors were analyzed among stratified factors such as sex, age, cell differentiation, etc. After assessing the clinical relevance of ATG4B and pATG4B protein expression level on the pathological stages and therapy with overall survival in patients with CRC, we proposed that ATG4B might serves as a biomarker for CRC, suggestive of a therapeutic target for the treatment of CRC.

## Materials and methods

### Immunohistochemistry (IHC)

118 tumor tissues of CRC were obtained from Kaohsiung Chang Gung Memorial Hospital as described previously [30]. The study was approved by the Institutional Review Board according to the Declaration of Helsinki (201600132B0). In short, tumor specimens were fixed with 10% formalin solution and embedded in paraffin. The proteins were probed with primary antibodies against ATG4B (dilution 1:100; A2981, Sigma-Aldrich, St. Louis, MO, USA) or pS383/392-ATG4B (dilution 1:100; homemade from phosphor-peptide immunized rabbit). Immunoblotting results with ATG4B silenced CRC cells confirmed the specificity of ATG4B antibody (Fig. 6A). The specificity of antibody against pS383/392-ATG4B were initially validated by Dot Blot assay using various concentration of phospho-S383 (ERFFDpSEDEDFEILSLC) and phospho-S392 peptide (ERFFDSEDEDFEILpSLC), respectively (data not shown). Moreover, we have reported that the antibody can recognize HEK293T cells expressing wild-type ATG4B, but not ATG4B S383/392A mutant [31], suggesting that our antibody is sufficient to tell the difference between S383/392 phosphorylated and non-phosphorylated ATG4B. The staining slides were further counterstained with hematoxylin. The colored immunohistochemistry (IHC) staining signals were acquired by Ultra Vision Quanto Detection System kits (Thermo Fisher Scientific, Fremont, CA, USA). The protein levels were scored by a pathologist according to intensities (0–3) and percentage (5% increment). The H-score index was determined by intensity multiply percentage, ranging 0-300. The cutoff for high and low expression of ATG4B or pS383/392-ATG4B was based on the receiver operating characteristic (ROC) curve.

### Cell culture and transfection

CRC HCT116 cells were purchased from Bioresource Collection and Research Ceter (BCRC, Taiwan) and maintained in Dulbecco’s Modified Eagle’s Medium (DMEM) (Invitrogen, 12100-046) along with 10% fetal bovine serum and antibiotics, including penicillin (100 U/ml), and streptomycin (100 mg/ml). The cells were reversely transfected with 10 nM scrambled siRNA (Life Technologies, 12935-112) or siRNA against ATG4B (Life Technologies, 20,218, s23245, s23246) using RNAiMAX (Life Technologies, 13778-150) as delivery reagent. The transfected cells were harvested to determine knockdown efficiency with immunoblotting using antibodies against ATG4B (dilution 1:100; A2981, Sigma-Aldrich, St. Louis, MO, USA) as described previously [8, 28].

### Cell mobility assays

Wound healing assays were used to access cell migratory activity with IBIDI Culture-Inserts (80,209, 35 mm, USA). The ATG4B silenced cells (2 × 10^5^ cells) were seeded into an insert for 16 h and removed for cell migration. The healing distances were quantified and measured with image J and tabulated with Prism 5 (GraphPad) using scramble siRNA transfected cells as control. Moreover, 70 µl cells (10^6^ cells/ml) were cultured with DMEM medium containing 1% FBS into 0.5% Matrigel coated transwell (657,638, Greiner Bio-One, UK). The invaded cells on the bottom side of the insert chamber were fixed with 3.7% paraformaldehyde and stained with 2% crystal violet. The stained cell colonies were imaged under a microscopy (490042-0002-000, ZEISS Axioscope, Carl Zeiss, München, Germany) and counted for cell invasion ability with image J software (National Institutes of Health, USA).

### Sphere Culture and Live/ Dead assay

The HCT116 cells (4000 cells/well) were transfected with 10 nM siRNA in an 96-well U-shaped bottomed plate (176,925, ultra-low attachment, Costar®, USA) for 3 days. The tumorspheres were treated with chemotherapeutic drug irinotecan (CPT, 1 µM, S1198, Selleckchem, Houston, TX, USA) or oxaliplatin (OXI, 10 µM, S1224, Selleckchem, Houston, TX, USA) for 48 h. The cell permeable calcein AM (1 µM) and non-permeable dye ethidium homodimer-1 (EthD-1, 2 µM) (L3224, LIVE/DEAD® Viability/Cytotoxicity Kit, ThermoFisher Scientific) were used to stain living and dead cells, respectively. The tumorspheres without treatment were defined as 100% living cells, while the tumorspheres treated with 0.1% saponin for 10 min were defined as 100% dead cells. The tumorspheres were imaged under a fluorescence microscopy (490042-0002-000, ZEISS Axioscope, Carl Zeiss, München, Germany). The living/dead staining cells were measured with excitation at 485 nm and emissions at 530 and 645 nm in a reader (Fluoroskan Ascent FL, Thermo Fisher Scientific).

### Statistical analysis

The gene expression for clinical relevance of CRC was obtained from The Cancer Genome Atlas (TCGA) database. The gene and protein expression were divided into high and low groups according to receiver operating characteristic curve (ROC). The *ATG4B* gene levels and protein levels of ATG4B and pS383/392-ATG4B lined to cumulative survival curves of CRC patients were analyzed with Kaplan-Meier method with the log-rank test. Multivariate Cox regression model with adjustment for cell differentiation (moderate + poor vs. well) and AJCC pathological stage (stage III + IV vs. stage I + II) was used to evaluate the correlation of ATG4B/pS383/392-ATG4B protein levels with overall survival or disease-free survival. A two-sided value of *p* < 0.05 was considered as statistically significant. For cell culture experiments, the significant results were calculated by a non-parametric 2-tailed Student’s *t*-test from three independent experiments.

## Results

### The relationship of ATG4B or pS383/392-ATG4B protein levels with overall and disease-free survival of CRC

**Fig. 1:**
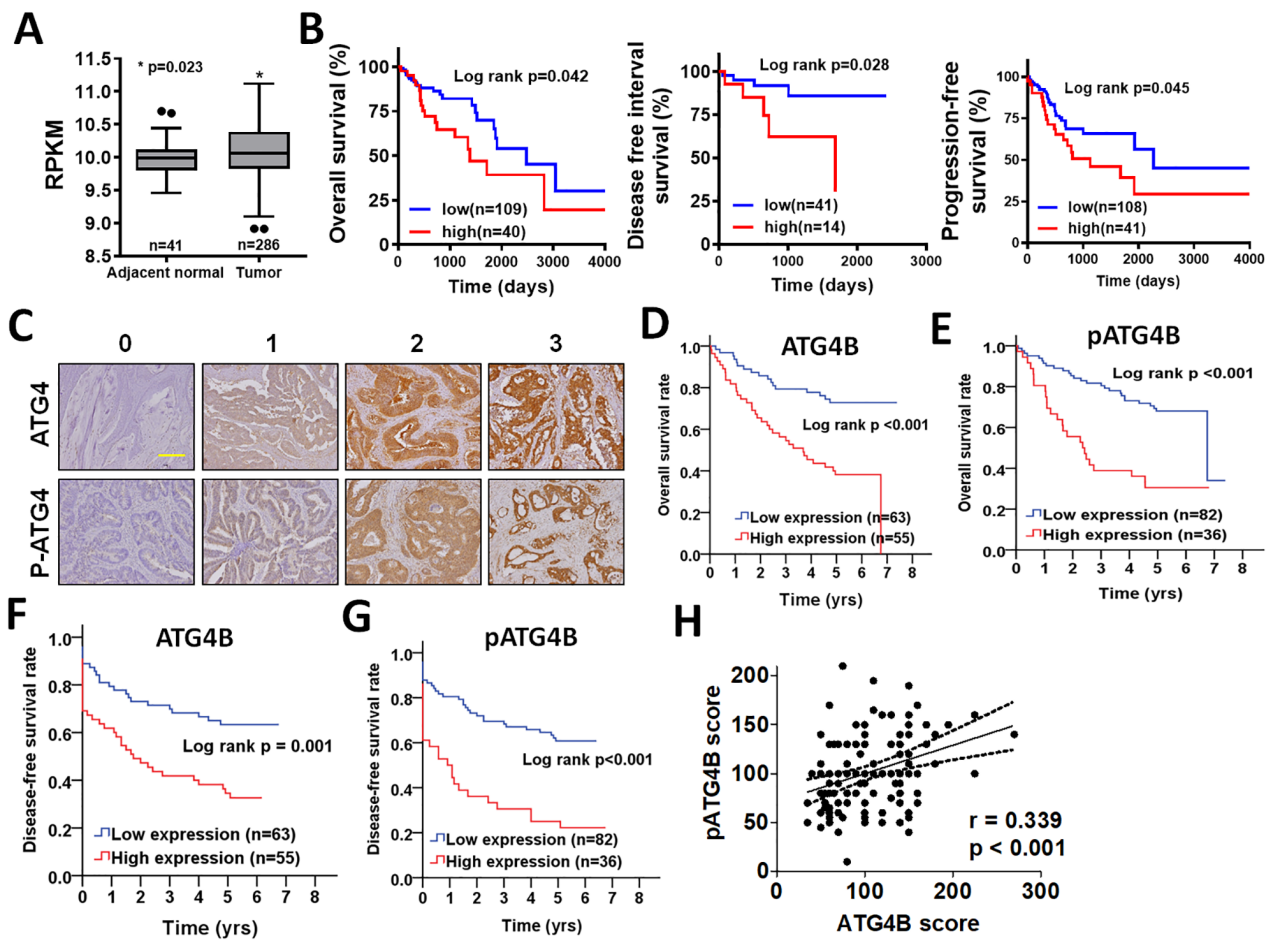
**The association of ATG4B and phosphorylated ATG4B with overall survival and DFS in patients with CRC. (A)** Gene expression levels of ATG4B in tumor and adjacent normal tissues were obtained from TCGA and expressed as Reads Per Kilobase per Million (RPKM). * *p* < 0.05 vs. adjacent normal tissues. **(B)** The ATG4B expression was divided into high and low groups according to receiver operating characteristic curve (ROC). The association of ATG4B expression with overall, disease-free and progression-free survival was analyzed by Kaplan-Meier plots. **(C)** The scoring for protein levels of ATG4B and its phosphorylated form (p-ATG4B) was determined according to staining intensity of immunohistochemistry as representative images. Scale bar: 200 μm. **(D)** Kaplan-Meier plots were used to analyze the correlation of ATG4B or **(E)** phosphorylated ATG4B (p-ATG4B) with overall survival of CRC patients. **(F)** Kaplan-Meier analysis was used to evaluate to the correlation of ATG4B or **(G)** phosphorylated ATG4B (p-ATG4B) with DFS of CRC patients was examined. **(H)** The correlation of ATG4B and p-ATG4B was analyzed by Pearson _X_2 test

Since ATG4B and its proteolytic activity are involved in the autophagy machinery and proliferation of cancer cells, the clinical correlation of ATG4B gene expression with CRC patients was initially analyzed according to TCGA database. *ATG4B* gene expression was significantly higher in tumor tissues of CRC patients compared with those in adjacent normal tissues (Fig. 1A). High levels of *ATG4B* gene expression were correlated with worse overall, disease-free, and progression-free survival of male patients (Fig. 1B), whereas it had no association with poor prognosis in female patients and any other clinicopathologic factors (supplementary Table S1). Moreover, clinical association of ATG4B and active form (pS383/392-ATG4B) in CRC patients was examined with IHC staining [25, 32]. The staining intensity was used to categorize the protein levels of ATG4B and p-S383/392-ATG4B as shown in standard staining slides (Fig. 1C). High levels of ATG4B and p-S383/392-ATG4B were positively correlated with worse overall survival compared to CRC patients with low protein levels of ATG4B (p < 0.001, Fig. 1D) and p-S383/392-ATG4B (p < 0.001, Fig. 1E). Similarly, high levels of both ATG4B (p = 0.001, Fig. 1F) and pS383/392-ATG4B (p = 0.001, Fig. 1G).

After adjustments on cell differentiation and AJCC stage, multiple Cox regression analyses results showed that high ATG4B and pS383/392-ATG4B had high risk of mortality, including overall survival (ATG4B: AHR: 5.71, p < 0.001; pS383/392-ATG4B: AHR: 2.50, p = 0.004, Table 1) and disease-free survival (ATG4B: AHR = 5.08, p < 0.001; pS383/392-ATG4B: AHR = 2.07, p = 0.011, Table 1). Moreover, ATG4B protein level is positively correlated with pS383/392-ATG4B (r = 0.339, p < 0.001, Fig. 1H). High levels of both ATG4B and pS383/392-ATG4B greatly increased the risk of mortality in both overall survival and disease-free survival of CRC patients (overall survival: AHR = 8.83, p < 0.001; disease-free survival: AHR = 6.50, p < 0.001, Table 1). These results suggest that both ATG4B and its active form pS383/392-ATG4B might be associated with cancer progression of CRC.

**Table 1:** Impact of expression levels on survival by the different demographic and clinicopathologic factors with colorectal cancer

Variable	ROC	No. (%)	CHR (95% CI)	*p value*	AHR (95% CI)	*p value*
**Overall survival**						
ATG4B	Low	63 (53.4)	1.00		1.00	
	High	55 (46.6)	3.13(1.75–5.59)	**< 0.001**	5.71(3.04–10.70)	**< 0.001**
Phospho-ATG4B	Low	82 (69.5)	1.00		1.00	
	High	36 (30.5)	3.04(1.75–5.26)	**< 0.001**	2.50(1.34–4.65)	**0.004**
ATG4B (L), p-ATG4B (L)		48 (40.7)	1.00		1.00	
ATG4B (H), p-ATG4B (L)		34 (28,8)	1.04 (0.58–1.87)	0.907^**a**^	2.25 (1.04–4.85)	0.039^c^
ATG4B (L), p-ATG4B (H)		15 (12.7)	0.81 (0.34–1.90)	0.622^**a**^	1.80 (0.66–4.93)	0.251^c^
ATG4B (H), p-ATG4B (H)		21 (17.8)	5.74 (3.18-10.338)	**< 0.001** ^**a**^	8.83 (4.13–18.90)	**< 0.001** ^**c**^
**Disease-free survival**						
ATG4B	Low	63 (53.4)	1.00		1.00	
	High	55 (46.6)	2.32(1.37–3.91)	**0.002**	5.08(2.79–9.22)	**< 0.001**
p-ATG4B	Low	82 (69.5)	1.00		1.00	
	High	36 (30.5)	2.89(1.73–4.84)	**< 0.001**	2.07(1.18–3.63)	**0.011**
ATG4B (L), p-ATG4B (L)		48 (40.7)	1.00		1.00	
ATG4B (H), p-ATG4B (L)		34 (28,8)	0.82 (0.46–1.46)	0.502^**a**^	1.50 (0.75–3.01)	0.249^c^
ATG4B (L), p-ATG4B (H)		15 (12.7)	0.87 (0.40–1.92)	0.733^**a**^	1.54 (0.63–3.75)	0.341^c^
ATG4B (H), p-ATG4B (H)		21 (17.8)	5.18 (2.95–9.11)	**< 0.001** ^**a**^	6.50 (3.29–12.84)	**< 0.001** ^**c**^

*Abbreviations: SCC, squamous cell carcinoma; CHR, crude hazard ratio; CI, confidence interval; AHR, adjusted hazard ratio; AJCC, American Joint Committee on Cancer; RT, radiotherapy.*

^a^ *p values were estimated by Cox’s regression.*

^b^ *p values were adjusted for cell differentiation (moderate + poor vs. well) and AJCC pathological stage (stage III + IV vs. stage I + II) by multivariate Cox’s regression.*

^c^ *p values were estimated by multivariate Cox’s regression.*

### The relationship of demographic, clinicopathologic factors and radiation therapy on ATG4B-mediated poor overall survival

**Fig. 2:**
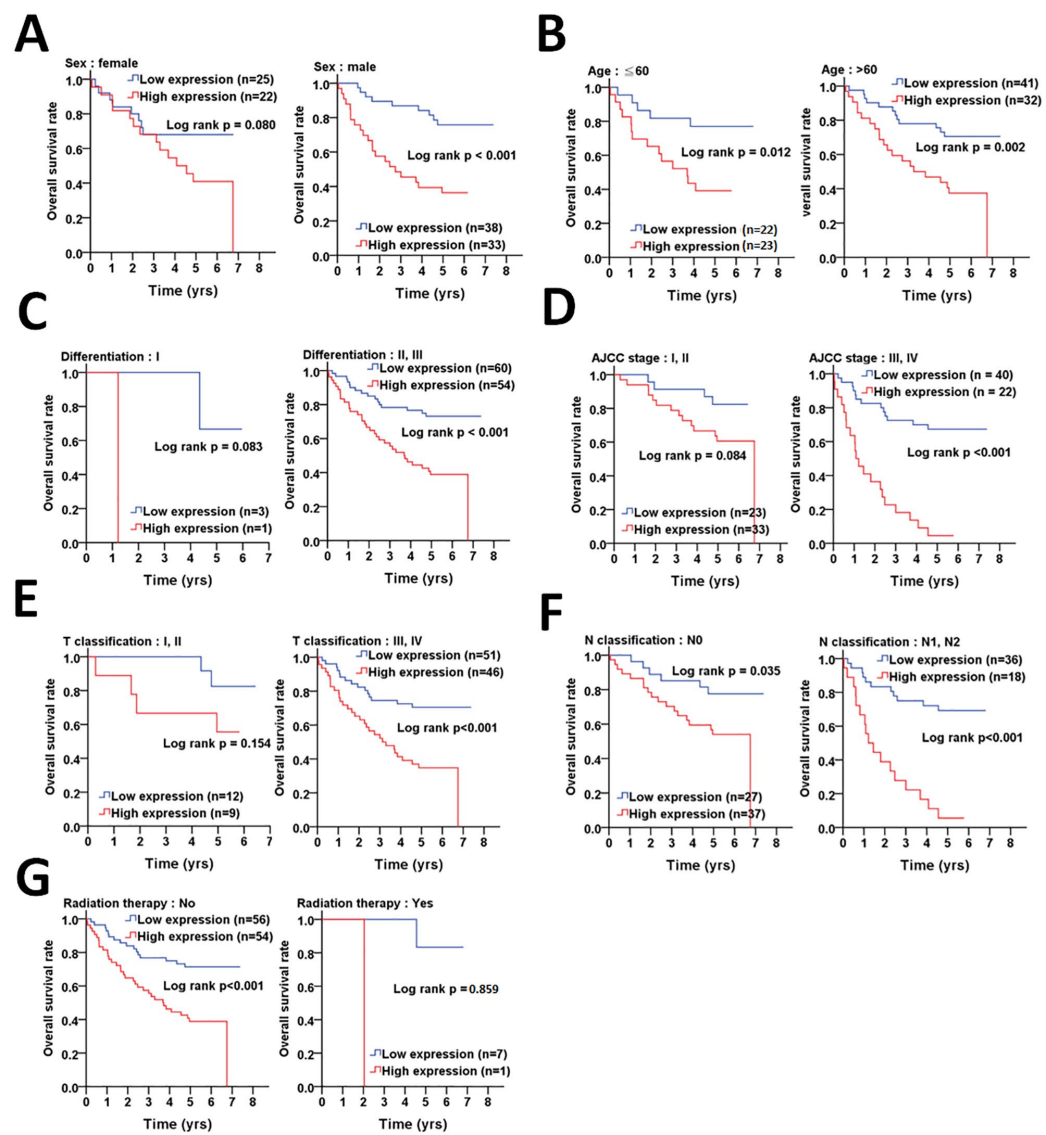
**The association of ATG4B with demographic, clinicopathologic factors and radiation therapy on overall survival in CRC patients. (A)** Kaplan-Meier plots were used to determine the association of demographic factors with ATG4B on overall survival, including gender and **(B)** age (< or > 60 years old). The association of ATG4B with clinicopathologic factors on overall survival was further inspected, such as **(C)** differentiation (I: well, II: moderate and III: poor), **(D)** clinicopathologic staging AJCC (I + II and III + IV), **(E)** T stages (tumor size, I + II and III + IV) and **(F)** N stages (lymph nodes invasion, 0 and 1 + 2). **(G)** The association of ATG4B with radiation therapy on overall survival was also evaluated. The significance of ATG4B protein levels on survival is shown with log rank

To inspect if correlation of ATG4B or pS383/392-ATG4B with poor overall survival rely on demographic factors or in certain clinicopathologic stages or in response to radiation therapy, a Kaplan-Meier curve was initially used for analysis (Fig. 2). High ATG4B was significantly linked to unfavorable overall survival in male patients with CRC (male: p < 0.001; female: p = 0.08, Fig. 2A), while it was correlated with poor overall survival in CRC patients with both age ≤ 60 (p = 0.012, Fig. 2B) and > 60 (p = 0.002). Moreover, high ATG4B protein levels was strongly associated with worse overall survival in CRC patients with poor cellular differentiation (p < 0.001, Fig. 2C) and advanced stages (AJCC stages III + IV: p < 0.001, Fig. 2D; T stages III + IV: p < 0.001, Fig. 2E). Nevertheless, there was significant correlation with overall survival in CRC patients with (N1 + N2, p = 0.001, Fig. 2F) or without (N0, p = 0.035, Fig. 2F) lymph node invasion. High ATG4B also had strong association with short overall survival of CRC patients who did not take (p < 0.001, Fig. 2G) radiation therapy. Consistently, analysis with adjustment of cell differentiation and AJCC stage showed that high ATG4B expression was significantly associated with high risk of mortality in CRC patients with demographic features, such as gender (males: AHR = 6.59, p < 0.001, Table 2; female: AHR = 3.69, p = 0.007), age (> 60:AHR = 6.74, p < 0.001; ≤ 60: AHR = 4.94, p = 0.003), and patients with advanced clinicopathological stages, including moderate/poor cell differentiation (AHR = 5.90, p < 0.001, Table 2), AJCC stage III + IV (AHR: 6.14, p < 0.001), T stage 3 + 4 (AHR = 4.73, p < 0.001), N stage 1 + 2 (AHR = 6.02, p < 0.001). In contrast, elevated ATG4B had a higher risk of mortality in patients who did not take radiotherapy (AHR = 4.88, p < 0.001), but had no significance in patients who took radiotherapy - likely due to the small cohort number in this subgroup.

**Table 2:** Impact of ATG4B expression levels on overall survival by the different demographic and clinicopathologic factors with colorectal cancer

Variable		No. (%)	CHR (95% CI)	p value*	AHR (95% CI)	*p value* ^†^
Sex						
Female	Low	25 (53.5)	1.00		1.00	
	High	22 (46.5)	2.14 (0.90–5.10)	0.087	3.69 (1.43–9.53)	**0.007** ^**a**^
Male	Low	38 (53.2)	1.00		1.00	
	High	33 (46.8)	3.92 (1.79–8.58)	**0.001**	6.59 (2.81–15.46)	**< 0.001** ^**a**^
Age, yrs						
≦ 60	Low	22 (48.9)	1.00		1.00	
	High	23 (51.1)	3.45 (1.24–9.60)	**0.018**	4.94 (1.72–14.24)	**0.003** ^**a**^
>60	Low	41 (56.2)	1.00		1.00	
	High	32 (43.8)	2.94 (1.44–5.98)	**0.003**	6.74 (2.83–16.01)	**< 0.001** ^**a**^
Cell differentiation						
Well	Low	3 (68.0)	1.00		1.00	
	High	1 (32.0)	434.45 (0.00-8.954E + 12)	0.616	434.45 (0.00-8.954E + 12)	0.616^b^
Moderate, poor	Low	60 (44.1)	1.00		1.00	
	High	54 (55.9)	3.07 (1.69–5.58)	**< 0.001**	5.90 (3.07–11.32)	**< 0.001** ^**b**^
AJCC pathological stage						
I, II	Low	23 (41.1)	1.00		1.00	
	High	33 (58.9)	2.60 (0.85–7.97)	0.096	3.00 (0.85–10.54)	0.087^c^
III, IV	Low	40 (64.5)	1.00		1.00	
	High	22 (35.5)	6.18 (3.00-12.72)	**< 0.001**	6.14 (2.97–12.70)	**< 0.001** ^**c**^
T classification						
T1, T2	Low	12 (57.1)	1.00		1.00	
	High	9 (42.9)	3.22 (0.59–17.64)	0.178	5.50 (0.61–49.32)	0.128^d^
T3, T4	Low	51 (52.6)	1.00		1.00	
	High	46 (47.4)	3.01 (1.62–5.59)	**< 0.001**	4.73 (2.40–9.31)	**< 0.001** ^**d**^
N classification						
N0	Low	27 (42.2)	1.00		1.00	
	High	37 (57.8)	2.60 (1.03–6.56)	**0.043**	2.65 (0.97–7.25)	0.059^e^
N1, N2	Low	36 (66.7)	1.00		1.00	
	High	18 (33.3)	6.10 (2.77–13.43)	**< 0.001**	6.02 (2.71–13.35)	**< 0.001** ^**e**^
Postoperative RT						
No	Low	56 (50.9)	1.00		1.00	
	High	54 (49.1)	2.83 (1.56–5.14)	**0.001**	4.88 (2.59–9.21)	**< 0.001** ^**a**^
Yes	Low	7 (87.5)	1.00		1.00	
	High	1 (12.5)	22097.29 (0.00-1.528E + 052)	0.859	22097.29 (0.00-1.528e + 052)	0.859^a^

*Abbreviations: SCC, squamous cell carcinoma; CHR, crude hazard ratio; CI, confidence interval; AHR, adjusted hazard ratio; AJCC, American Joint Committee on Cancer; RT, radiotherapy.*

*p values were estimated by Cox’s regression.

^†^ *p values were estimated by multivariate Cox’s regression.*

^*a*^ *Adjusted for cell differentiation (moderate + poor vs. well) and AJCC pathological stage (stage III + IV vs. stage I + II).*

^*b*^ *Adjusted for AJCC pathological stage (stage III + IV vs. stage I + II).*

^*c*^ *Adjusted for cell differentiation (moderate + poor vs. well).*

^*d*^ *Adjusted for cell differentiation (moderate + poor vs. well) and N classification (N1, N2 vs. N0).*

^*e*^ *Adjusted for cell differentiation (moderate + poor vs. well) and T classification (T3, T4 vs. T1 + T2).*

**Table 3:** Impact of pS383/392-ATG4B expression levels on overall survival by the different demographic and clinicopathologic factors with colorectal cancer

Variable		No. (%)	CHR (95% CI)	p value*	AHR (95% CI)	*p value* ^†^
Sex						
Female	Low	35 (74.5)	1.00		1.00	
	High	12 (25.5)	1.98 (0.82–4.78)	0.127	1.03 (0.37–2.90)	0.953^a^
Male	Low	47 (66.2)	1.00		1.00	
	High	24 (33.8)	4.04 (1.94–8.40)	**< 0.001**	3.72 (1.70–8.14)	**0.001** ^**a**^
Age, years						
≦ 60	Low	29 (64.4)	1.00		1.00	
	High	16 (35.6)	3.73 (1.49–9.35)	**0.005**	2.30 (0.79–6.65)	0.125^a^
>60	Low	53 (72.6)	1.00		1.00	
	High	20 (27.4)	2.74 (1.36–5.49)	**0.005**	2.43 (1.14–5.21)	**0.022** ^**a**^
Cell differentiation						
Well	Low	3 (75.0)	1.00		1.00	
	High	1 (25.0)	434.45 (0.00-8.954E + 12)	0.616	92.86 (0.00-13265331940)	0.636^b^
Moderate, poor	Low	79 (69.3)	1.00		1.00	
	High	35 (30.7)	2.93 (1.67–5.13)	**< 0.001**	2.26 (1.27-4.00)	**0.005** ^**b**^
AJCC pathological stage						
I, II	Low	50 (89.3)	1.00		1.00	
	High	6 (10.7)	4.74 (1.53–14.71)	**0.007**	4.81 (1.53–15.09)	**0.007** ^**c**^
III, IV	Low	32 (51.6)	1.00		1.00	
	High	30 (48.4)	2.12 (1.06–4.24)	**0.034**	2.07 (1.03–4.16)	**0.042** ^**c**^
T classification						
T1, T2	Low	21 (100.0)	1.00		1.00	
	High	0 (0.0)	Incalculable		Incalculable	
T3, T4	Low	61 (62.9)	1.00		1.00	
	High	36 (37.1)	2.82 (1.57–5.07)	**0.001**	2.86 (1.51–5.42)	**0.001** ^**d**^
N classification						
N0	Low	55 (85.9)	1.00		1.00	
	High	9 (14.1)	5.38 (2.17–13.36)	**< 0.001**	5.32 (1.98–14.27)	**0.001** ^**e**^
N1, N2	Low	27 (50.0)	1.00		1.00	
	High	27 (50.0)	2.19 (1.01–4.76)	**0.047**	2.12 (0.97–4.64)	0.060^e^
Postoperative RT						
No	Low	79 (71.8)	1.00		1.00	
	High	31 (28.2)	3.90 (2.22–6.87)	**< 0.001**	3.02 (1.63–5.62)	**< 0.001** ^**a**^
Yes	Low	3 (37.5)	1.00		1.00	
	High	5 (62.5)	0.35 (0.02–6.15)	0.470	14.53 (0.00-326310532.6)	0.757^a^

*Abbreviations: SCC, squamous cell carcinoma; CHR, crude hazard ratio; CI, confidence interval; AHR, adjusted hazard ratio; AJCC, American Joint Committee on Cancer; RT, radiotherapy.*

*p values were estimated by Cox’s regression.

^†^ *p values were estimated by multivariate Cox’s regression.*

^*a*^ *Adjusted for cell differentiation (moderate + poor vs. well) and AJCC pathological stage (stage III + IV vs. stage I + II).*

^*b*^ *Adjusted for AJCC pathological stage (stage III + IV vs. stage I + II).*

^*c*^ *Adjusted for cell differentiation (moderate + poor vs. well).*

^*d*^ *Adjusted for cell differentiation (moderate + poor vs. well) and N classification (N1, N2 vs. N0).*

^*e*^ *Adjusted for cell differentiation (moderate + poor vs. well) and T classification (T3, T4 vs. T1 + T2).*

**Fig. 3:**
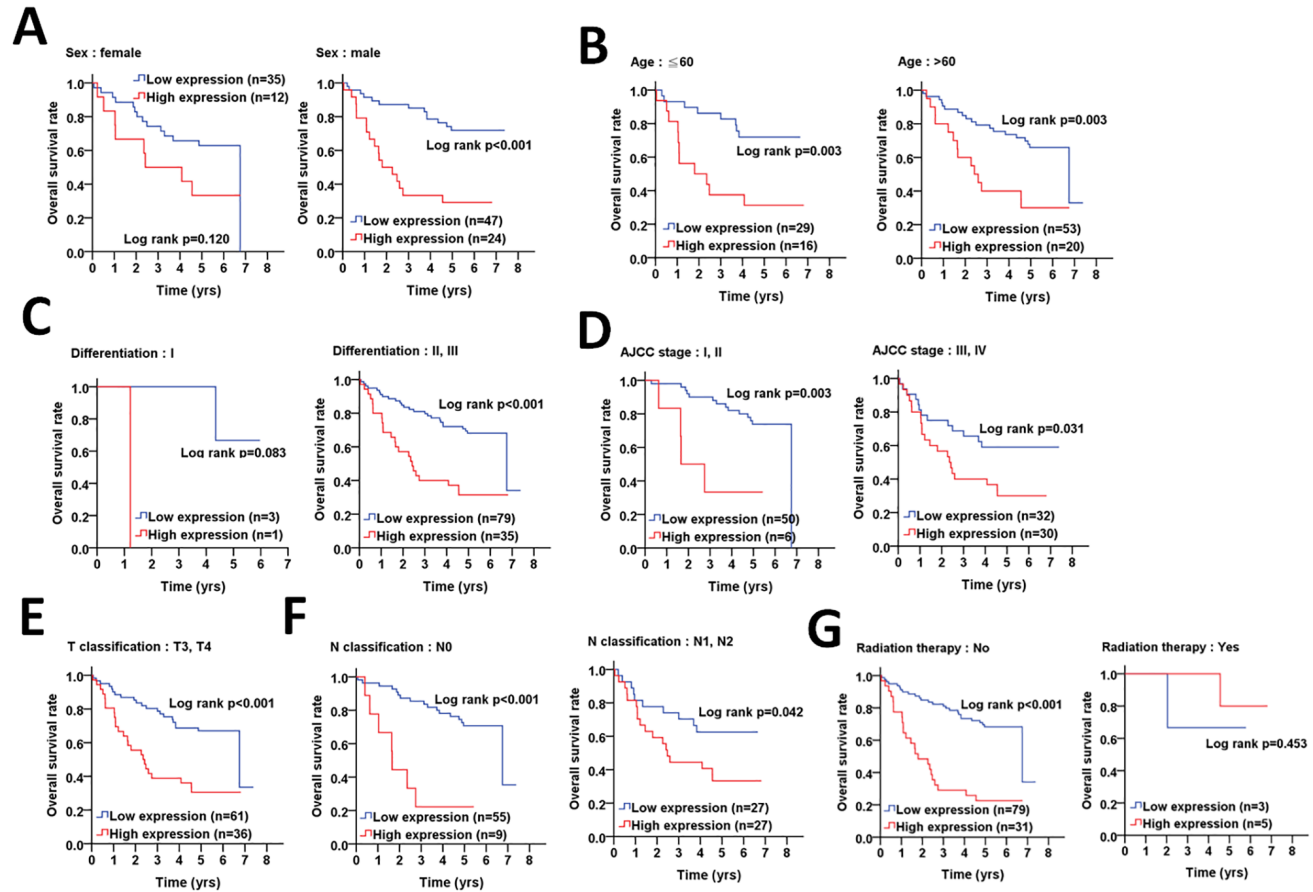
**The association of phosphorylated ATG4B with demographic, clinicopathologic factors and radiation therapy on overall survival in colorectal cancer patients. (A)** Kaplan-Meier plots were used to determine the association of demographic factors with phosphorylated ATG4B (pS383/392-ATG4B) on overall survival, including gender and **(B)** age (< or > 60 years old). The association of pS383/392-ATG4B with clinicopathologic factors on overall survival was further inspected, such as **(C)** differentiation (I: well, II: moderate and III: poor), **(D)** clinicopathologic staging AJCC (I + II and III + IV), **(E)** T stages (tumor size, III + IV) and **(F)** N stages (lymph nodes invasion, 0 and 1 + 2). **(G)** The association of pS383/392-ATG4B with radiation therapy on overall survival was also evaluated. The significance of pS383/392-ATG4B protein levels on survival is shown with log rank

Likewise, the significant correlation of high pS383/392-ATG4B protein level with unfavorable overall survival was observed in male CRC patients (p = < 0.001, Fig. 3A), different ages (≤ 60: p = 0.003, > 60: p = 0.003, Fig. 3B), cell differentiation (moderate + poor(II + III): p < 0.001, Fig. 3C), AJCC stages (I + II: p = 0.003, III + IV: p = 0.031, Fig. 3D), T stage T3 + T4 (p < 0.001, Fig. 3E), N stage (N0: p < 0.001; N1 + N: p = 0.042, Fig. 3F) and patients without radiation therapy (p < 0.001, Fig. 3G). In contrast, multiple Cox regression analyses revealed that high levels of pS383/392-ATG4B had higher risk of mortality in CRC patients with early clinicopathological stages (AJCC stage I + II: AHR = 4.81, p = 0.007; N0 stage: AHR = 5.32, p = 0.001; No radiation therapy: AHR = 3.02, p = 0.001, Table 3).

### Correlation of the ATG4B and pS383/392-ATG4B protein levels with recurrence of CRC

**Fig. 4:**
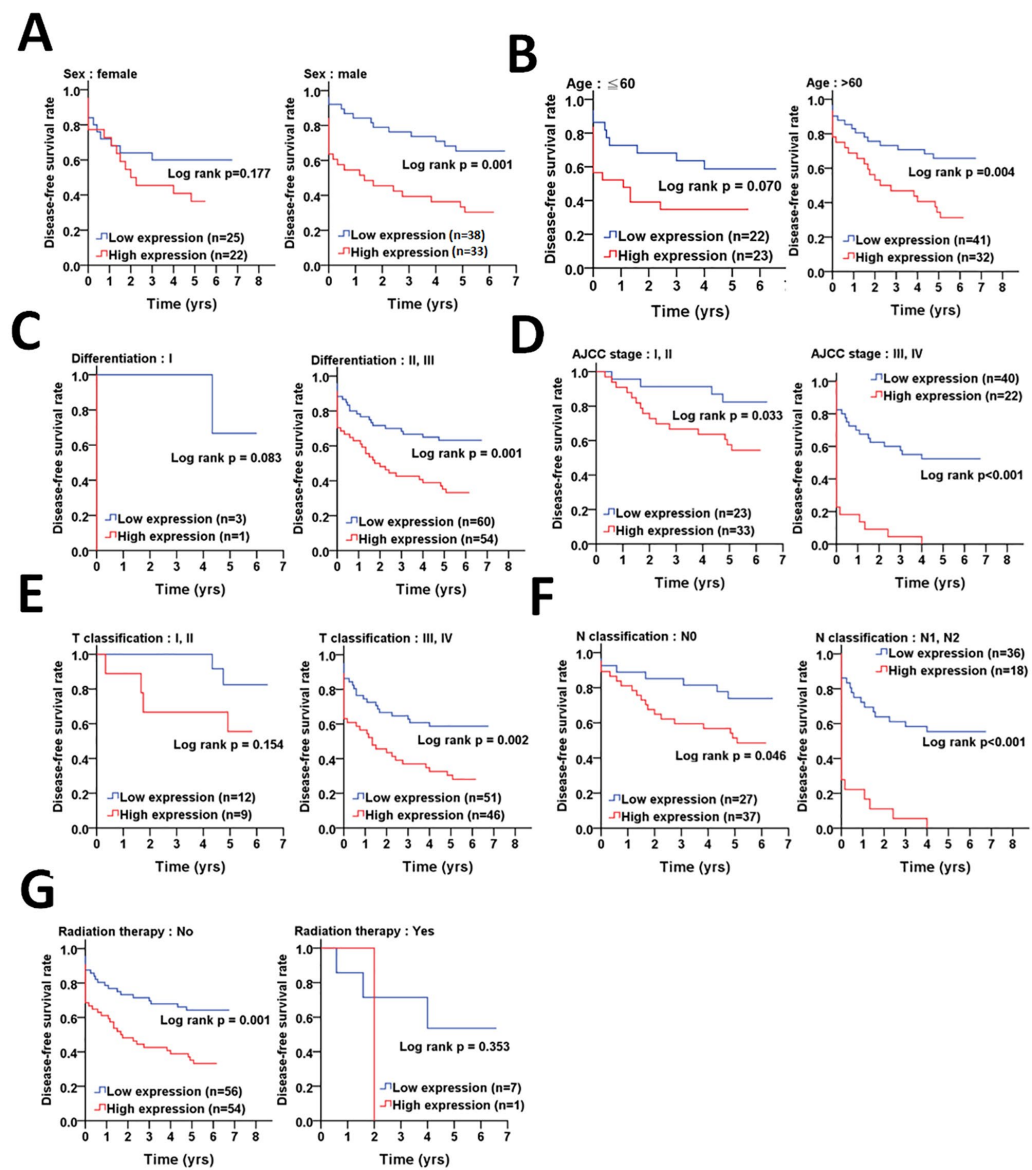
**The association of ATG4B with demographic, clinicopathologic factors and radiation therapy on disease-free survival in colorectal cancer patients. (A)** Kaplan-Meier plots were used to determine the association of demographic factors with ATG4B on disease-free survival, including gender and **(B)** age (< or > 60 years old). The association of ATG4B with clinicopathologic factors on disease-free survival was further inspected, such as **(C)** differentiation (I: well, II: moderate and III: poor), **(D)** clinicopathologic staging AJCC (I + II and III + IV), **(E)** T stages (tumor size, I + II and III + IV) and **(F)** N stages (lymph nodes invasion, 0 and 1 + 2). **(G)** The association of ATG4B with radiation therapy on disease-free survival was also evaluated. The significance of ATG4B protein levels on disease-free survival is shown with log rank

To evaluate if ATG4B and pS383/392-ATG4B are correlated with relapse of CRC, Kaplan-Meier curve revealed that high ATG4B had worsened disease-free survival in males (p < 0.001) and elderly (p = 0.004) CRC patients (Fig. 4A and B). Similar to outcomes from overall survival, high ATG4B protein levels were associated with unfavorable disease-free survival, particularly in CRC patients with advanced stages, such as cell differentiation moderate + poor II + III: p < 0.001, AJCC stages III + IV: p < 0.001; T stage T3 + T4: p = 0.002; N stage N1 + N2: p < 0.001 and patients who did not receive radiation therapy (p = 0.001) (Fig. 4). After adjustment with pathological stages and cell differentiation, high ATG4B protein levels had worsened disease-free survival in males (AHR = 6.78, p < 0.001, Table 4) and elderly patients (AHR = 5.62, p < 0.001). High ATG4B was positively related to elevated mortality in patients with advanced tumors, including differentiated tumor cells (moderate and poor: AHR = 5.17, p < 0.001, Table 4), AJCC stage III + IV (AHR = 4.37, p < 0.001), large tumor size T3 + T4 (AHR = 3.73, p < 0.001); lymph node invasion N1 + N2 stage (AHR = 4.69, p = 0.001). Nevertheless, high ATG4B was correlated with shorter disease-free survival in patients without taking radiation therapy (AHR = 4.67, p = 0.001, Table 4).

**Table 4:** Impact of ATG4B expression levels on disease-free survival by the different demographic and clinicopathologic factors with colorectal cancer

Variable		No. (%)	CHR (95% CI)	p value*	AHR (95% CI)	*p value* ^†^
Sex						
Female	Low	25 (53.5)	1.00		1.00	
	High	22 (46.5)	1.70 (0.76–3.84)	0.199	3.18 (1.30–7.80)	**0.012** ^**a**^
Male	Low	38 (53.2)	1.00		1.00	
	High	33 (46.8)	2.79 (1.41–5.54)	**0.003**	6.78 (2.89–15.88)	**< 0.001** ^**a**^
Age, yrs						
≦ 60	Low	22 (48.9)	1.00		1.00	
	High	23 (51.1)	2.01 (0.87–4.61)	0.102	3.55 (1.41–8.94)	**0.007** ^**a**^
>60	Low	41 (56.2)	1.00		1.00	
	High	32 (43.8)	2.51 (1.28–4.92)	**0.007**	5.62 (2.50-12.66)	**< 0.001** ^**a**^
Cell differentiation						
Well	Low	3 (68.0)	1.00		1.00	
	High	1 (32.0)	434.45 (0.00-8.954E + 12)	0.616	434.45 (0.00-8.954E + 12)	0.616^b^
Moderate, poor	Low	60 (44.1)	1.00		1.00	
	High	54 (55.9)	2.24 (1.31–3.81)	**0.003**	5.17 (2.80–9.54)	**< 0.001** ^**b**^
AJCC pathological stage						
I, II	Low	12 (57.1)	1.00		1.00	
	High	9 (42.9)	3.11 (1.03–9.38)	**0.044**	3.60 (1.04–12.46)	**0.043** ^**c**^
III, IV	Low	51 (52.6)	1.00		1.00	
	High	46 (47.4)	4.41 (2.24–8.68)	**< 0.001**	4.37 (2.21–8.65)	**< 0.001** ^**c**^
T classification						
T1, T2	Low	12 (57.1)	1.00		1.00	
	High	9 (42.9)	3.22 (0.59–17.64)	0.178	5.50 (0.61–49.32)	0.128^d^
T3, T4	Low	51 (52.6)	1.00		1.00	
	High	46 (47.4)	2.18 (1.26–3.78)	**0.005**	3.73 (2.00-6.98)	**< 0.001** ^**d**^
N classification						
N0	Low	27 (42.2)	1.00		1.00	
	High	37 (57.8)	2.33 (0.98–5.56)	0.056	2.18 (0.86–5.53)	0.102^e^
N1, N2	Low	36 (66.7)	1.00		1.00	
	High	18 (33.3)	4.75 (2.29–9.86)	**< 0.001**	4.69 (2.24–9.80)	**< 0.001** ^**e**^
Postoperative RT						
No	Low	56 (50.9)	1.00		1.00	
	High	54 (49.1)	2.32 (1.34–4.02)	**0.003**	4.67 (2.53–8.64)	**< 0.001** ^**a**^
Yes	Low	7 (87.5)	1.00		1.00	
	High	1 (12.5)	2.96 (0.27–32.84)	0.376	2.96 (0.27–32.84)	0.376^a^

*Abbreviations: SCC, squamous cell carcinoma; CHR, crude hazard ratio; CI, confidence interval; AHR, adjusted hazard ratio; AJCC, American Joint Committee on Cancer; RT, radiotherapy.*

*p values were estimated by Cox’s regression.

^†^ *p values were estimated by multivariate Cox’s regression.*

^*a*^ *Adjusted for cell differentiation (moderate + poor vs. well) and AJCC pathological stage (stage III + IV vs. stage I + II).*

^*b*^ *Adjusted for AJCC pathological stage (stage III + IV vs. stage I + II).*

^*c*^ *Adjusted for cell differentiation (moderate + poor vs. well).*

^*d*^ *Adjusted for cell differentiation (moderate + poor vs. well) and N classification (N1, N2 vs. N0).*

^*e*^ *Adjusted for cell differentiation (moderate + poor vs. well) and T classification (T3, T4 vs. T1 + T2).*

**Fig. 5:**
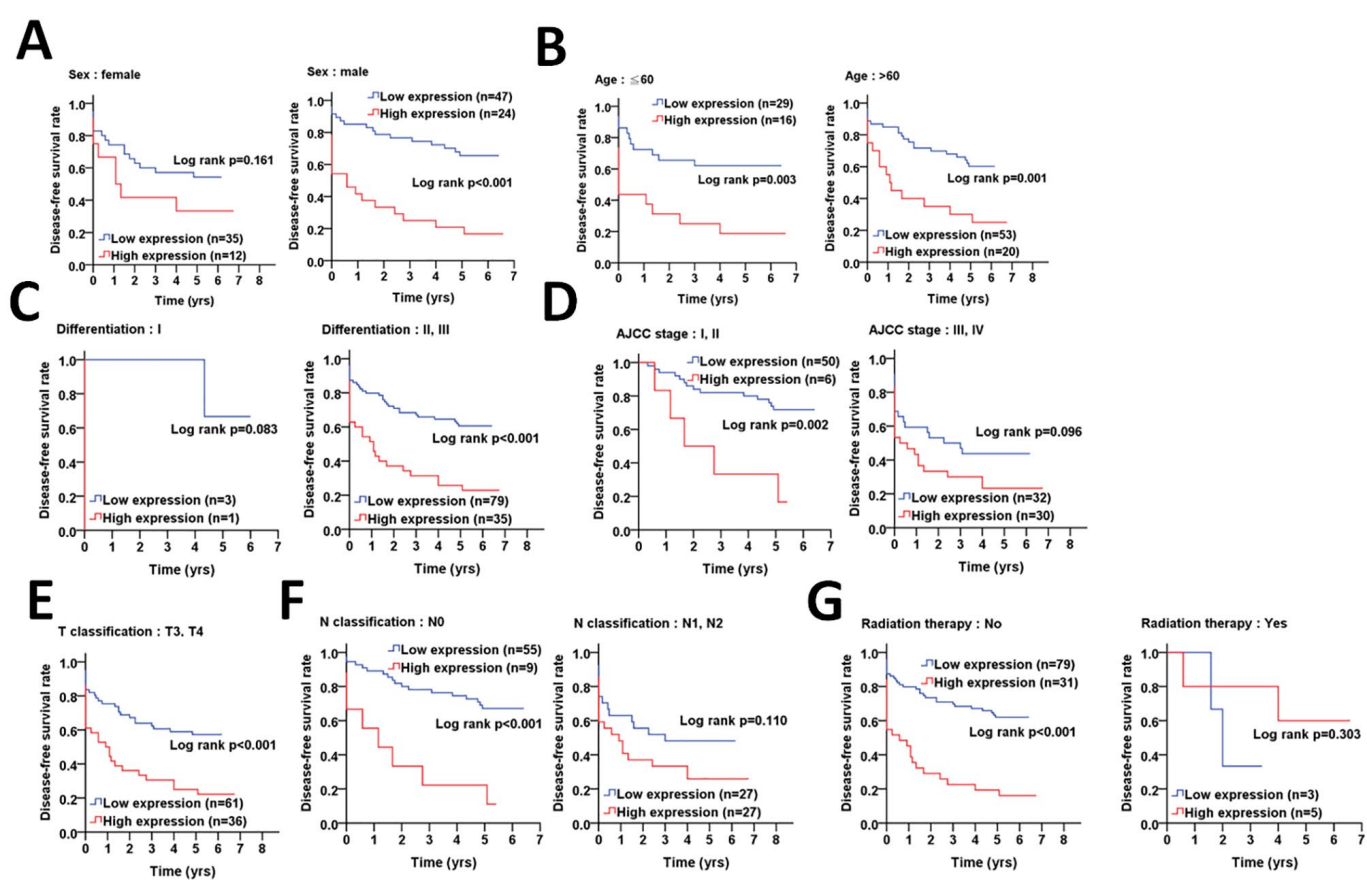
**The association of phosphorylated ATG4B with demographic, clinicopathologic factors and radiation therapy on disease-free survival in colorectal cancer patients. (A)** Kaplan-Meier plots were used to determine the association of demographic factors with phosphorylated ATG4B (pS383/392-ATG4B) on disease-free survival, including gender and **(B)** age (< or > 60 years old). The association of pS383/392-ATG4B with clinicopathologic factors on disease-free survival was further inspected, such as **(C)** differentiation (I: well, II: moderate and III: poor), **(D)** clinicopathologic staging AJCC (I + II and III + IV), **(E)** T stages (tumor size, III + IV) and **(F)** N stages (lymph nodes invasion, 0 and 1 + 2). **(G)** The association of pS383/392-ATG4B with radiation therapy on disease-free survival was also evaluated. The significance of pS383/392-ATG4B protein levels on survival is shown with log rank

On the other hand, Kaplan-Meier curve indicated that high protein levels of pS383/392-ATG4B were associated with shorter disease-free survival (Fig. 5), particularly in males (p < 0.001, Fig. 5A), and patients with early stages, such as AJCC stage I and II (p = 0.002, Fig. 5D) and lymph node invasion stage N0 (p < 0.001, Fig. 5F), and patients who did not receive radiation therapy (p < 0.001, Fig. 5G).

Similarly, Cox regression analysis showed that higher protein levels of pS383/392-ATG4B increased the risk of mortality in disease-free survival of males (AHR: 3.37, p = 0.001, Table 5), the elderly (AHR: 2.35, p = 0.019, Table 5), and patients with early clinicopathological stages (AJCC stage I + II: AHR = 4.59, p = 0.004; N0 stage: AHR = 4.38, p = 0.001; No radiation therapy: AHR = 2.51, p = 0.002, Table 5). However, elevated levels of pS383/392-ATG4B was correlated with high risk of mortality in patients with undifferentiated (moderate/poor: AHR = 2.02, p = 0.015, Table 5) and larger tumors (T3 and T4 stages: AHR = 2.01, p = 0.013, Table 5).

**Table 5:** Impact of pS383/392-ATG4B expression levels on disease-free survival by the different demographic and clinicopathologic factors with colorectal cancer

Variable		No. (%)	CHR (95% CI)	p value*	AHR (95% CI)	*p value* ^†^
Sex						
Female	Low	35 (74.5)	1.00		1.00	
	High	12 (25.5)	1.78 (0.76–4.85)	0.184	0.75 (0.29–1.94)	0.549^a^
Male	Low	47 (66.2)	1.00		1.00	
	High	24 (33.8)	4.00 (2.04–7.85)	**< 0.001**	3.37 (1.67–6.81)	**0.001** ^**a**^
Age, years						
≦ 60	Low	29 (64.4)	1.00		1.00	
	High	16 (35.6)	2.96 (1.31–6.68)	**0.009**	1.39 (0.58–3.37)	0.464^a^
>60	Low	53 (72.6)	1.00		1.00	
	High	20 (27.4)	2.76 (1.41–5.39)	**0.003**	2.35 (1.15–4.78)	**0.019** ^**a**^
Cell differentiation						
Well	Low	3 (75.0)	1.00		1.00	
	High	1 (25.0)	434.45 (0.00-8.954E + 12)	0.616	Incalculable	
Moderate, poor	Low	79 (69.3)	1.00		1.00	
	High	35 (30.7)	2.78 (1.65–4.69)	**< 0.001**	2.02 (1.15–3.56)	**0.015** ^**b**^
AJCC pathological stage						
I, II	Low	50 (89.3)	1.00		1.00	
	High	6 (10.7)	4.55 (1.63–12.76)	**0.004**	4.59 (1.62–12.98)	**0.004** ^**c**^
III, IV	Low	32 (51.6)	1.00		1.00	
	High	30 (48.4)	1.58 (0.85–2.94)	0.147	1.55 (0.83–2.89)	0.172^c^
T classification						
T1, T2	Low	21 (100.0)	1.00		1.00	
	High	0 (0.0)	Incalculable		Incalculable	
T3, T4	Low	61 (62.9)	1.00		1.00	
	High	36 (37.1)	2.46 (1.43–4.21)	**0.001**	2.01 (1.16–3.48)	**0.013** ^**d**^
N classification						
N0	Low	55 (85.9)	1.00		1.00	
	High	9 (14.1)	4.92 (2.10–11.50)	**< 0.001**	4.38 (1.76–10.91)	**0.001** ^**e**^
N1, N2	Low	27 (50.0)	1.00		1.00	
	High	27 (50.0)	1.65 (0.83–3.28)	0.151	1.61 (0.80–3.21)	0.180^e^
Postoperative RT						
No	Low	79 (71.8)	1.00		1.00	
	High	31 (28.2)	3.51 (2.05–6.01)	< 0.001	2.51 (1.41–4.46)	**0.002** ^**a**^
Yes	Low	3 (37.5)	1.00		1.00	
	High	5 (62.5)	0.30 (0.03–3.36)	0.331	0.38(0.02–6.10)	0.490^a^

*Abbreviations: SCC, squamous cell carcinoma; CHR, crude hazard ratio; CI, confidence interval; AHR, adjusted hazard ratio; AJCC, American Joint Committee on Cancer; RT, radiotherapy.*

*p values were estimated by Cox’s regression.

^†^ *p values were estimated by multivariate Cox’s regression.*

^*a*^ *Adjusted for cell differentiation (moderate + poor vs. well) and AJCC pathological stage (stage III + IV vs. stage I + II).*

^*b*^ *Adjusted for AJCC pathological stage (stage III + IV vs. stage I + II).*

^*c*^ *Adjusted for cell differentiation (moderate + poor vs. well).*

^*d*^ *Adjusted for cell differentiation (moderate + poor vs. well) and N classification (N1, N2 vs. N0).*

^*e*^ *Adjusted for cell differentiation (moderate + poor vs. well) and T classification (T3, T4 vs. T1 + T2).*

### The biological functions of ATG4B in the cell viability and mobility of CRC cells

**Fig. 6:**
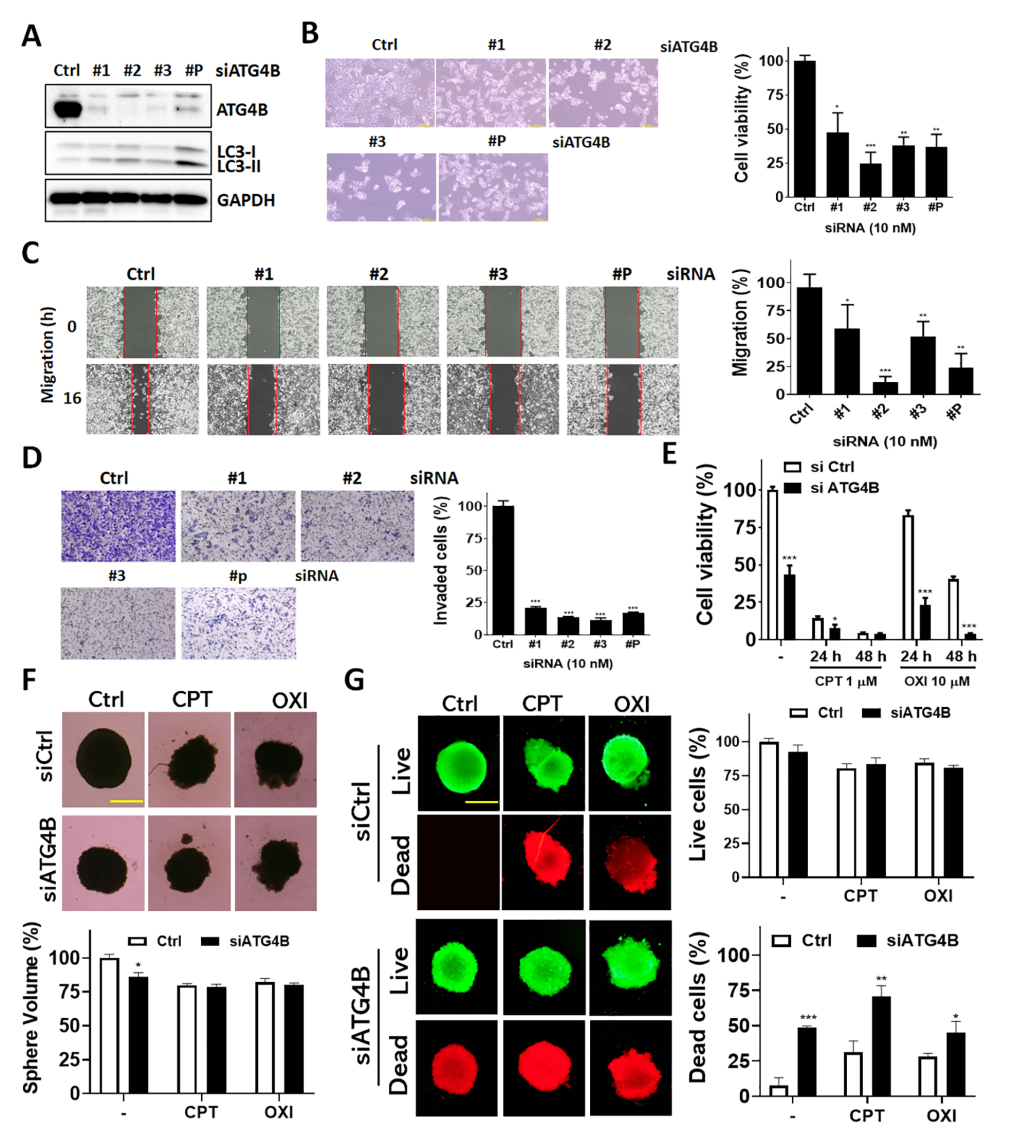
**Effects of silencing ATG4B on mobility and chemosensitivity in human colorectal cancer cells. (A)** HCT116 cells were transfected with 10 nM scramble siRNA (siCtrl) or three individual siRNA against ATG4B (siATG4B #1, #2 and #3) or pooled siRNA (#P) against ATG4B for 72 h and examine knockdown efficiency by immunoblotting. **(B)** The ATG4B silenced cells were observed in left panel and measured for cell viability by CellTiter Glo. **(C)** ATG4B silenced CRC cells were cultured in migration insert and **(D)** Transwell chamber to quantify the migratory distance and invaded cells, respectively. **(E)** CRC cells were silenced with 10 nM scramble or siRNA against ATG4B for 48 h and treated with chemotherapeutic drug irinotecan (CPT, 1 µM) or oxaliplatin (OXI, 10 µM) for 24 or 48 h. The cell viability was measured with CellTiter Glo. **(F)** The siRNA transfected CRC cells were cultured as spheres and treated with chemotherapeutic drug (CPT, 1 µM, OXI, 10 µM) for 2 days as images. The tumor spheres were quantified compared to control sphere. Scale bar: 400 μm. **(G)** The sphere viability was monitored by LIVE/DEAD staining dyes. The fluorescence was read and quantified. * *p* < 0.05, ** *p* < 0.01, *** *p* < 0.001

Given the aforementioned clinical results, elevated protein levels of ATG4B and pS383/392-ATG4B, worsens survival rate in CRC, suggesting ATG4B might be involved in tumor malignancy. To further inspect the role of ATG4B in CRC cells, human colorectal cancer cells were silenced by individual or pooled siRNA against ATG4B for cell viability and mobility assay (Fig. 6A-D). Knockdown of ATG4B with either individual or pooled siRNA largely suppressed protein level of ATG4B (Fig. 6A and supplementary Figure S1) and significantly reduced cell density and viability (Fig. 6B). The ability to migrate and invade was also diminished in ATG4B silenced CRC cells (Fig. 6C and D), consistent with the clinical results. In addition, high ATG4B and its active form pS383/392-ATG4B had worsened disease-free survival, implying ATG4B might be critical to therapeutic resistance and relapse. We further examined the effects of silencing ATG4B in CRC cells treated with chemotherapeutic drugs, such as oxaliplatin and CPT (Fig. 6E-G). Knockdown of ATG4B enhanced chemotherapeutic agents-inhibited cell viability (Fig. 6E). To further mimic tumor 3D structure and examine the cell viability effects, siRNA transfected CRC cells were treated with chemotherapeutic drugs in tumorsphere culture model. The size of tumorspheres of ATG4B silenced or chemotherapeutic agents treated cells were slightly decreased (Fig. 6F). The dead cells population in chemotherapeutic drugs treated cells and silencing ATG4B also significantly enhanced the cytotoxicity of chemotherapeutic drugs (Fig. 6G), consistent with the notion that we observed in clinical settings.

## Discussion

Autophagy is intimately associated with cancer development and malignancy. The role of autophagy in CRC is still controversial, and may serve as a tumor suppressor or tumor promoter [33]. ATG4 (ATG4A, ATG4B, ATG4C and ATG4D), a cysteine protease family, plays a crucial role in autophagy signaling and correlated with cancer progression in different types of cancers [22, 24, 28]. However, little is known about ATG4 in clinical outcomes of CRC cancer. ATG4B is a functional dominant protease among ATG4 family to promote autophagy activity [21, 34, 35]. Limited cohort study (n = 20) shows that ATG4B protein level is elevated in tumor parts of CRC patients [28]. Emerging studies also indicate that ATG4B silencer or inhibitor reduces cell proliferation and metastatic features of cancer cells [26, 27, 29, 36]. Perhaps then, ATG4B may play its role as a tumor promoter in cancer. We report the following findings: First, the higher protein levels of both ATG4B and its active form pS383/392-ATG4B worsens overall survival and disease-free survival. Second, high ATG4B levels contributed to higher risk of mortality in patients with advanced pathological stages, while pS383/392-ATG4B was correlated with poor survival in patients with early pathological stages. Third, silencing ATG4B attenuated cell viability and mobility, enhanced chemosensitivity of CRC cells.

Phosphorylation of many ATG proteins have been reported to modulate autophagy activity [37]. Phosphorylation of ATG9a induces autophagy, whereas phosphorylation of ATG14 inhibits autophagy [38]. However, phosphorylation on different sites in ATG proteins can have opposing effects on autophagy. For example, the phosphorylation of ULK1 (ATG1) at Thr180, Ser317, Ser467 promotes autophagy. In contrast, phosphorylation at Ser469, Ser495, Ser533 leads to reduced autophagy activity [37]. Similarly, phosphorylation of ATG4B at Ser34, Ser383, Ser392 increase ATG4B proteolytic activity and enhances binding with LC3 and autophagic activity in cells [32, 39, 32, 40]. Nevertheless, phosphorylation at Ser316 decreases ATG4B and autophagy activity [41]. Our present study indicated that both ATG4B and its active form pS383/392-ATG4B were elevated in tumor cells of CRC patients compared to adjacent normal cells. In contrast to single protein level of either ATG4B or pS383/392-ATG4B, high co-expression of ATG4B and pS383/392-ATG4B had much higher mortality risk in CRC. Thus, pS383/392-ATG4B levels may imply increase autophagy activity in tumor cells, which strongly suggests that autophagy might facilitate CRC development and tumor malignancy. Besides phosphorylation, there are several post-translational modifications of ATG4B, such as O-GlcNAcylation, oxidation, S-Nitrosylation and ubiquitination, to modulate autophagy [42]. Nevertheless, other ATG proteins or regulators are likely to also influence overall autophagic activity. However, the role of autophagy in CRC cancer prognosis requires further study and clarity.

In a stratified survival analysis of ATG4B and pS383/392-ATG4B, there was increased risk of cancer death in CRC patients with early clinicopathological stages, such as AJCC stage I and II and no lymph node invasion (N0), whereas there were no significant effects in patients with well differentiated tumor cells and small tumor sizes (T1 and T2). Perhaps this observation can be attributed to the low case number (well differentiation: n = 1), or no (T1 + T2: n = 0) in high level group of pS383/392-ATG4B in these classifications. Consequently, there is an urgent need to expand the cohort size for further interpretation of the clinical association at the various stages in CRC. Similarly, due to low case numbers in CRC patients who received radiation therapy, we were unable to ascertain a meaningful correlation of ATG4B and pS383/392-ATG4B in the context of radiation resistance. Moreover, radiotherapy was only treated for rectal cancer patients, which would inevitably require a larger patient cohort to examine the involvement of ATG4B or pS383/392-ATG4B in the prognosis of rectal cancer.

ATG4B serves as a tumor promoter in various types of cancers, where elevated expression is associated with worsened survival of cancer patients [22]. The literature reports emerging studies that are focused on the development of several ATG4B inhibitors to block cancer cell proliferation, mobility, and drug resistance. In particular, the natural product Azalomycin is used for treating gastric cancers, while a small molecule inhibitor S130, a Chinese herb extract, have been used for CRC and oral cancer cells (Fig. 4a) [29, 36, 43]. It is also worthwhile mentioning that the anti-fungal drug tioconazole has been repurposed for various types of solid tumors as well.

In line with our current results, ATG4B and its active form pS383/392-ATG4B are highly associated with poor survival of CRC patients. Silencing ATG4B attenuated cell growth, mobility, and synergized cancer cells to chemotherapeutic drugs. There is no specific inhibitor to block pS383/392-ATG4B in cells. Thus, we transfected expression vector encoding wild-type ATG4B, S383/392A mutant or C74A catalytic mutant in HCT116 cells (data not shown). The cell viability of cells with ATG4B mutants was slightly decreased, likely due to low transfection efficiency or little amount of endogenous wild-type ATG4B can cover autophagy signaling in cells. Taken together, these results suggest that pharmacological inhibition of ATG4B might shed a light to treat CRC, at least in ATG4B over-activated CRC. Nevertheless, autophagy inhibition not only impedes cancer cell viability, but also play dual roles in immune cells by either enhancing or disrupting their cytotoxic activity [44], which requires further determination for the justification of ATG4B inhibitors in cancer treatment.

## Conclusion

Our results might provide ATG4B and pS383/392-ATG4B as new prognostic biomarkers for CRC patients. Moreover, ATG4B could serve as a therapeutic target candidate for CRC therapy.

## Electronic supplementary material

Below is the link to the electronic supplementary material.

Supplementary Material 1

Supplementary Material 2

## Data Availability

The datasets supporting the conclusions of this article are included within the article.

## Abbreviations

ATG: autophagy-related
ATG4B: autophagy-related 4B, cysteine peptidase
CQ: chloroquine
LC3: microtubule-associated protein 1 light chain 3
CRC: Colorectal cancer
