# Supplementary figures and images for: ATG4B and pS383/392-ATG4B serve as potential biomarkers and therapeutic targets of colorectal cancer
Source: Cancer Cell Int. 2023 Apr 10;23:63. doi: 10.1186/s12935-023-02909-7 (PMC10088137; doi:10.1186/s12935-023-02909-7)

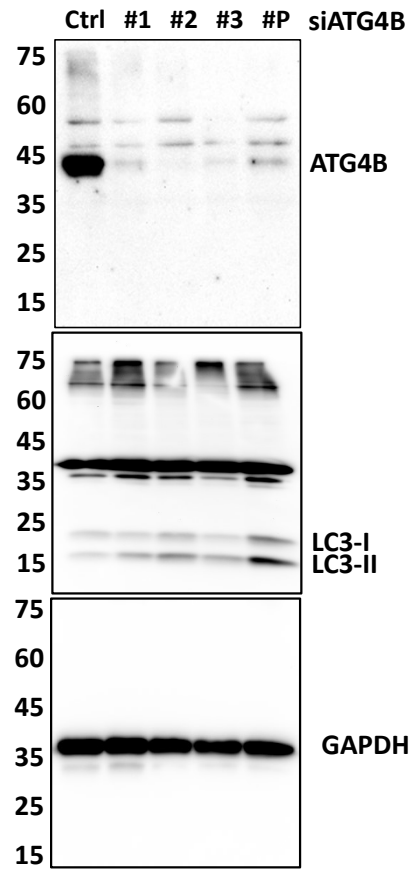

Supplement: Supplementary file 1 — Supplementary Material 1 [file 12935_2023_2909_MOESM1_ESM.pdf]
