# Supplementary material for: ATG4B and pS383/392-ATG4B serve as potential biomarkers and therapeutic targets of colorectal cancer
Source: Cancer Cell Int. 2023 Apr 10;23:63. doi: 10.1186/s12935-023-02909-7 (PMC10088137; doi:10.1186/s12935-023-02909-7)
Supplement: Supplementary file 2 — Supplementary Material 2 [file 12935_2023_2909_MOESM2_ESM.pdf]

Table S1. Impact of ATG4B expression levels on **overall survival** by the different demographic and clinicopathologic factors with colorectal cancer

Variable	ROC	No. (%)	CHR (95% CI)	*p value**	AHR (95% CI)	*p value*†
Sex						
Female	Low	92 (75.4)	1		1	
	High	30 (24.6)	1.38 (0.57-3.34)	0.472	1.53 (0.63-3.72)	0.353[a]
Male	Low	109 (73.2)	1		1	
	High	40 (26.8)	1.03 (0.50-2.11)	**0.046**	1.69 (0.89-3.19)	0.108[a]
Age, yrs						
≦ 70	Low	120 (74.5)	1		1	
	High	41 (25.5)	1.59 (0.73-3.48)	0.248	1.65 (0.75-3.63)	0.214[a]
＞70	Low	81 (73.6)	1		1	
	High	29 (26.4)	1.84 (0.95-3.59)	0.072	1.56 (0.79-3.06)	0.201[a]
AJCC pathological stage						
I, II	Low	119 (78.3)	1			
	High	33 (21.7)	1.20 (0.49-2.92)	0.686		
III, IV	Low	82 (68.9)	1			
	High	37 (31.1)	1.71 (0.91-3.21)	0.094		
T classification						
T1, T2	Low	41 (87.2)	1		1	
	High	6 (12.8)	0.041 (0.00-256657.48)	0.689	0.00 (0.00-)	0.990[b]
T3, T4	Low	160 (71.4)	1		1	
	High	64 (28.6)	1.61 (0.96-2.71)	0.070	1.58 (0.94-2.65)	0.085[b]
N classification						
N0	Low	123 (78.3)	1		1	
	High	34 (21.7)	1.35 (0.58-3.12)	0.486	1.21 (0.52-2.82)	0.665[c]
N1, N2	Low	78 (68.4)	1		1	
	High	36 (31.6)	1.55 (0.81-2.97)	0.181	1.59 (0.83-3.05)	0.164[c]

Abbreviations: CHR, crude hazard ratio; CI, confidence interval; AHR, adjusted hazard ratio; AJCC, American Joint Committee on Cancer

**p values were estimated by Cox's regression.*

†*p values were estimated by multivariate Cox's regression.*

[a]*Adjusted for AJCC pathological stage (stage III+ IV vs stage I+II).*

[b]*Adjusted for N classification (N1, N2 vs N0).*

[c]*Adjusted for T classification (T3, T4 vs T1+T2).*

Table S1. Impact of ATG4B expression levels on **progression-free interval survival** by the different demographic and clinicopathologic factors with colorectal cancer

Variable	ROC	No. (%)	CHR (95% CI)	*p value**	AHR (95% CI)	*p value*†
Sex						
Female	Low	91 (74.6)	1		1	
	High	31 (25.4)	0.81 (0.35-1.90)	0.631	0.78 (0.33-1.81)	0.557[a]
Male	Low	108 (72.5)			1	
	High	41 (27.5)	1.78 (1.01-3.15)	**0.048**	1.53 (0.86-2.74)	0.150[a]
Age, yrs						
≦ 70	Low	118 (73.3)	1		1	
	High	43 (26.7)	1.11 (0.60-2.05)	0.746	1.01 (0.54-1.88)	0.967[a]
＞70	Low	81 (73.6)	1		1	
	High	29 (26.4)	1.84 (0.90-3.74)	0.094	1.62 (0.79-3.34)	0.188[a]
AJCC pathological stage						
I, II	Low	119 (78.3)	1			
	High	33 (21.7)	1.55 (0.72-3.32)	0.260		
III, IV	Low	80 (67.2)	1			
	High	39 (32.8)	1.06 (0.59-1.92)	0.844		
T classification						
T1, T2	Low	41 (87.2)	1		1	
	High	6 (12.8)	0.39 (0.00-2342.03)	0.564	0.00 (0.00-)	0.985[b]
T3, T4	Low	158 (70.5)	1		1	
	High	66 (29.5)	1.27 (0.79-2.05)	0.318	1.18 (0.73-1.90)	0.501[b]
N classification						
N0	Low	123 (78.3)	1		1	
	High	34 (21.7)	1.64 (0.79-3.38)	0.184	1.46 (0.70-3.04)	0.310[c]
N1, N2	Low	76 (66.7)	1		1	
	High	38 (33.3)	0.97 (0.53-1.79)	0.930	0.97 (0.52-1.78)	0.910[c]

Abbreviations: CHR, crude hazard ratio; CI, confidence interval; AHR, adjusted hazard ratio; AJCC, American Joint Committee on Cancer

**p values were estimated by Cox's regression.*

†*p values were estimated by multivariate Cox's regression.*

[a]*Adjusted for AJCC pathological stage (stage III+ IV vs stage I+II).*

[b]*Adjusted for N classification (N1, N2 vs N0).*

[c]*Adjusted for T classification (T3, T4 vs T1+T2).*

Table S1. Impact of ATG4B expression levels on **disease-free interval survival** by the different demographic and clinicopathologic factors with colorectal cancer

Variable	ROC	No. (%)	CHR (95% CI)	*p value**	AHR (95% CI)	*p value*†
Sex						
Female	Low	29 (72.5)	1		1	
	High	11 (27.5)	0.67 (0.07-6.12)	0.721	0.66 (0.07-6.02)	0.712[a]
Male	Low	41 (74.5)	1		1	
	High	14 (25.5)	3.95 (1.05-14.84)	**0.042**	3.98 (1.05-15.13)	**0.043**[a]
Age, yrs						
≦ 70	Low	46 (73.0)	1		1	
	High	17 (27.0)	1.77 (0.42-7.55)	0.441	1.72 (0.40-7.46)	0.466[a]
＞70	Low	24 (75.0)	1		1	
	High	8 (25.0)	4.09 (0.82-20.45)	0.086	4.20 (0.83-21.28)	0.083[a]
AJCC pathological stage						
I, II	Low	52 (76.5)	1			
	High	16 (23.5)	1.61 (0.40-6.49)	0.500		
III, IV	Low	18 (66.7)	1			
	High	9 (33.3)	3.51 (0.58-21.08)	0.171		
T classification						
T1, T2	Low	19 (76.0)	1		1	
	High	6 (24.0)	0.31 (0.00-20280.22)	0.611	0.31 (0.00-20280.22)	0.611[b]
T3, T4	Low	51 (72.9)	1		1	
	High	19 (27.1)	2.86 (0.92-8.92)	0.070	2.84 (0.90-8.99)	0.075[b]
N classification						
N0	Low	52 (77.6)	1		1	
	High	15 (22.4)	1.12 (0.22-5.55)	0.893	1.11 (0.22-5.49)	0.903[c]
N1, N2	Low	18 (64.3)	1		1	
	High	10 (35.7)	4.34 (0.79-23.87)	0.091	4.34 (0.79-23.87)	0.091[c]

Abbreviations: CHR, crude hazard ratio; CI, confidence interval; AHR, adjusted hazard ratio; AJCC, American Joint Committee on Cancer

**p values were estimated by Cox's regression.*

†*p values were estimated by multivariate Cox's regression.*

[a]*Adjusted for AJCC pathological stage (stage III+ IV vs stage I+II).*

[b]*Adjusted for N classification (N1, N2 vs N0).*

[c]*Adjusted for T classification (T3, T4 vs T1+T2).*
